# Trephining in a Case of General Paralysis

**Published:** 1892-01

**Authors:** 


### TREPHINING IN A CASE OF GENERAL PARALYSIS.

---

M. Key reported a case in which he thought the disease was benefited by the intervention of the surgeon. The dura mater presented no unusual appearance. Beneath the meninges the large vessels presented a gelatinous aspect and showed milk-like plates. The brain formed a hernia which filled up the opening in the skull. A rapid examination was made.

The incisions in the dura mater were there reunited by sutures, as were also the rents in the pericranium.

The operation, which lasted an hour, was performed under strict antiseptic precautions. On awakening, the patient was quiet and contented. Eight days later, the cicatrization of wound was completed and the patient returned to ward. A month and a half later he returned to his family, the states of depression and exaltation having disappeared.

M. Key remarks that cases of this kind may be benefited by the surgeon, even though there is no tumor or irritation of the brain, just as cases of symptomatic or essential epilepsy may be benefited. The incurability of general paresis and the recognized futility of internal treatment Justify this operation.—*Le Progres Medical*, August 15, 1891.

